# Non‐invasive sampling reveals low mitochondrial genetic diversity for an island endemic species: The critically endangered Grenada Dove *Leptotila wellsi*

**DOI:** 10.1002/ece3.10767

**Published:** 2023-11-23

**Authors:** Catherine Peters, Matthew Geary, Charlotte Hosie, Howard Nelson, Bonnie Rusk, Anna Muir

**Affiliations:** ^1^ Conservation Biology Research Group, Department of Biological Sciences University of Chester Chester UK; ^2^ University of Cambridge Cambridge UK; ^3^ Grenada Dove Conservation Programme St Georges Grenada

**Keywords:** Grenada Dove, island conservation, isolated populations, *Leptotila wellsi*, mitochondrial DNA

## Abstract

As an island endemic with a decreasing population, the critically endangered Grenada Dove *Leptotila wellsi* is threatened by accelerated loss of genetic diversity resulting from ongoing habitat fragmentation. Small, threatened populations are difficult to sample directly but advances in molecular methods mean that non‐invasive samples can be used. We performed the first assessment of genetic diversity of populations of Grenada Dove by (a) assessing mtDNA genetic diversity in the only two areas of occupancy on Grenada, (b) defining the number of haplotypes present at each site and (c) evaluating evidence of isolation between sites. We used non‐invasively collected samples from two locations: Mt Hartman (*n* = 18) and Perseverance (*n* = 12). DNA extraction and PCR were used to amplify 1751 bps of mtDNA from two mitochondrial markers: NADH dehydrogenase 2 (*ND2*) and Cytochrome b (*Cyt b*). Haplotype diversity (*h*) of 0.4, a nucleotide diversity (*π*) of 0.00023 and two unique haplotypes were identified within the *ND2* sequences; a single haplotype was identified within the *Cyt b* sequences. Of the two haplotypes identified, the most common haplotype (haplotype A = 73.9%) was observed at both sites and the other (haplotype B = 26.1%) was unique to Perseverance. Our results show low mitochondrial genetic diversity and clear evidence for genetically isolated populations. The Grenada Dove needs urgent conservation action, including habitat protection and potentially augmentation of gene flow by translocation in order to increase genetic resilience and diversity with the ultimate aim of securing the long‐term survival of this critically endangered species.

## Graphical abstract

We performed the first assessment of genetic diversity of populations of Grenada Dove by (a) assessing mtDNA genetic diversity in the only two areas of occupancy on Grenada, (b) defining the number of haplotypes present at each site and (c) evaluating evidence of isolation between sites. Our results show low mitochondrial genetic diversity, a non‐expanding population and clear evidence for genetically isolated populations. The Grenada Dove needs urgent conservation action, including habitat protection and potentially augmentation of gene flow by translocation in order to increase genetic resilience and diversity with the ultimate aim of securing the long‐term survival of this critically endangered species.

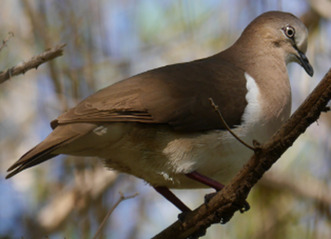

## 1 INTRODUCTION

Island populations are generally small and isolated, with their range restricted by the physical boundaries of the island they inhabit (Frankham et al., 2017; Groombridge et al., 2018). As such, low levels of genetic variability are often observed in island restricted populations (Hudson et al., 2000). Island populations are often formed by a small founder event and are more prone to bottlenecks, genetic drift, inbreeding depression and reduced genetic diversity (Dudaniec et al., 2011; Frankham, 1997; Gonzalez‐Quevedo et al., 2015; Grant et al., 2001) which, as a result, can reduce the viability of populations (Kahilainen et al., 2014). Conservation of genetic diversity is therefore imperative in ensuring the long‐term survival of a species, particularly in small and isolated populations, which are at an increased risk of loss of genetic diversity (Frankham et al., 2019; Lacy, 2000).

Obtaining samples such as tissue or blood from rare, cryptic or elusive species can be logistically difficult (Horváth et al., 2005; Mills et al., 2000). Moreover, for Endangered species with low encounter rates which are often sensitive to environmental disturbance, it can be difficult to obtain permits for more intrusive sampling methods, which in some cases are considered unethical (Segelbacher, 2002). Biological samples collected non‐invasively such as feathers, eggshell, faecal matter and shed skin can be collected in the field with minimal disturbance to the target species (Bohmann et al., 2014; Mills et al., 2000). However, species identification from non‐invasively collected samples is often difficult when working with species sharing the same habitat with similar somatic features (Ahlers et al., 2017; Coghlan et al., 2012; Waits & Paetkau, 2005). In addition, sample type, quality and condition can often not be controlled for, affecting the utility of such samples for genetic analysis; however, non‐invasive sampling may be the only opportunity to obtain samples for rare or elusive species (Mills et al., 2000; Peters et al., 2019). Given advances in non‐invasive techniques have demonstrated the usability of non‐invasive samples for species identification (Peters et al., 2019), phylogenetic analysis (Peters et al., 2022) and genotyping (Presti et al., 2013), it could, therefore, be argued that this type of sampling would be the preferred approach when taking into account ethical and welfare considerations, particularly for threatened species (Baus et al., 2019; Russello et al., 2015; Taberlet & Luikart, 1999).

**FIGURE 1:**
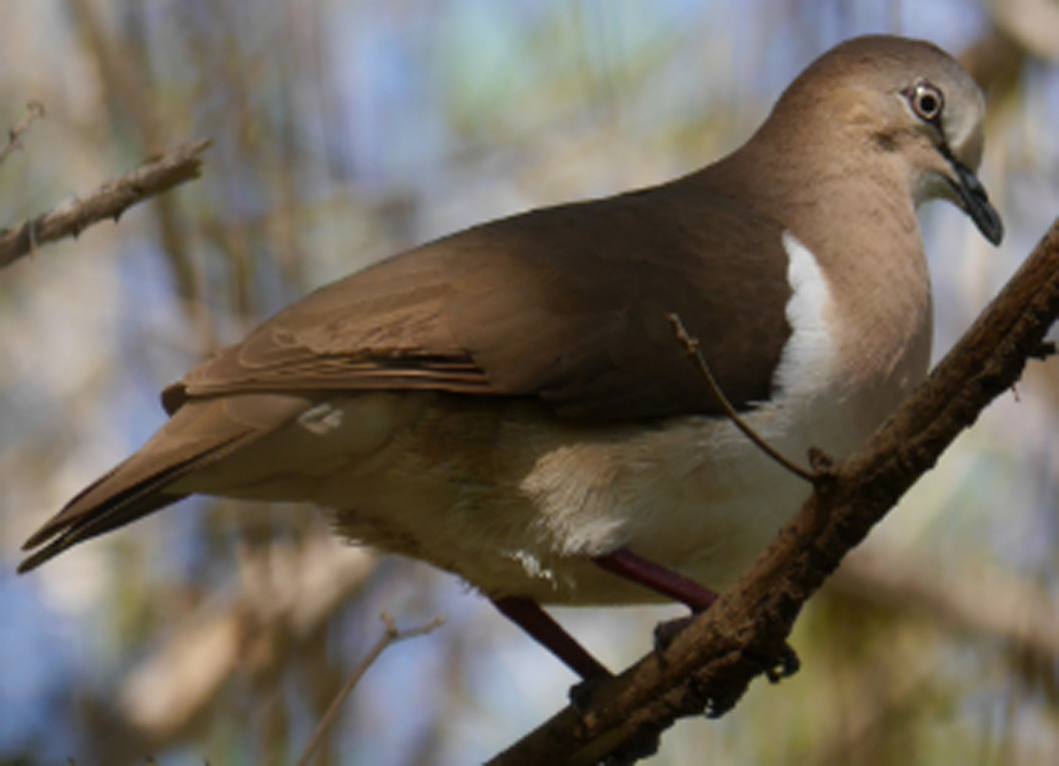
Grenada Dove *Leptotila wellsi*. Photo credit: Howard Nelson.

The Grenada Dove *Leptotila wellsi* (Figure 1) is a critically endangered (BirdLife International, 2021) species endemic to the island of Grenada (Peters et al., 2022; Rusk, 2017) with the most recent population survey estimating just 160 ± 30 individuals left of this species (Rivera‐Milán et al., 2015). The population is thought to be decreasing and it is estimated that it will see a 1%–19% reduction over the next three generations (BirdLife International, 2021). Its historical distribution was once widespread across dry coastal zones in the North‐East, West and South‐West of Grenada, including off‐shore islands (Baptista et al., 2020; Bond, 1973; Clarke, 1905; Lack & Lack, 1973). However, the Grenada Dove has experienced a severe reduction in population size and range with populations now presumed to be limited to sites in the West (Perseverance, Woodford and Beausejour area) and Southwest (Mount [Mt] Hartman Estate and surrounding area) of the Island which are approximately 1.6 km^2^ and 2.23 km^2^ respectively (Bolton et al., 2015; Rivera‐Milán et al., 2015; Rusk, 2009, 2017).

Although thought to be historically connected, these two remnant populations are now believed to be isolated due to the 9 km^2^ development—including the islands capital city St Georges—which separates the two (BirdLife International, 2021; Blockstein, 1991; Rusk, 2017). In addition to population isolation, the Grenada Dove is also threatened by habitat loss from industrial and commercial development such as road construction, housing and agricultural developments (Bolton et al., 2015; Rivera‐Milán et al., 2015; Rusk, 2017). A serious threat to the Grenada Dove population is a proposed resort construction at Mt Hartman estate and the surrounding area, which would drastically reduce available habitat in an area which holds approximately 48% of this species according to the census conducted in 2007 (Rusk, 2008, 2010).

Genetic variation in isolated populations is often lost over time as a result of long‐term genetic drift (Bollmer et al., 2005; Reynolds et al., 2015) and isolated populations pose a survival risk as they are more vulnerable to inbreeding and genetic erosion (Major et al., 2021; Wright et al., 2008). Isolation and fragmentation through habitat loss reduces population connectivity and results in lack of gene flow due to dispersal barriers (Cros et al., 2020; VanderWerf et al., 2010). Both dispersal patterns and population connectivity of the Grenada Dove are unclear (Baptista et al., 2020). In populations with low levels of connectivity and gene flow, alleles are easily lost to genetic drift, which are fundamental causes of loss of genetic diversity making populations more prone to extinction (Asai et al., 2006; Major et al., 2021). Given the small population (Rivera‐Milán et al., 2015; Rusk, 2017) and fragmented habitat (Bolton et al., 2015) of this critically endangered island species, isolated populations and lack of gene flow pose a large risk of inbreeding, genetic erosion and decreased survival. Therefore, investigation of genetic variation of this species is imperative to determine the most effective conservation strategies for this species (Major et al., 2021; Mischler et al., 2018).

Here, we assess the genetic diversity of the Grenada Dove across its range to inform evidence‐based conservation management at a population level. We used DNA extracted from non‐invasively collected feather samples from Grenada to (a) assess mtDNA genetic diversity of Grenada Dove in the two areas of occupancy, (b) define the number of haplotypes present in Grenada Dove at each site and (c) evaluate evidence of isolation between sites. We used this information to infer whether populations were isolated based on site‐specific haplotypes and to assess the level of genetic diversity both within and between these populations.

## 2 METHODS

### 2.1 Sample collection

Moulted feathers and eggshell samples from Grenada Dove were collected opportunistically in the months of May and July between 2014 and 2018 by members of the Forest and National Parks Department of the Government of Grenada from two known dove habitats: Mt Hartman Estate and Perseverance, Grenada. We used non‐invasive sampling methods to ensure minimal disturbance to this critically endangered species. Information such as site and date were recorded, and where possible the location using a handheld GPS, to accompany each sample. Feather samples (*n* = 194) and eggshell samples (*n* = 3) were stored at 4°C and imported to the United Kingdom using CITES import (546012/01, 567389/01) and export licences (Grenada IACUC 14002, 14004) in September 2016 and June 2018 where they were cleaned with 70% ethanol and stored at −20°C.

### 2.2 DNA extraction and PCR

DNA extraction was carried out with the QIAGEN DNeasy® Blood and Tissue kit (QIAGEN Inc., Crawley) using the optimised protocol outlined in Peters et al. (2019). Positive Grenada Dove identification was confirmed using species‐specific primers (Appendix S1). Samples displaying no species‐specific band were excluded from further investigation on the grounds that they were either poor quality or not from the target species. PCR was used to amplify mitochondrial markers: *ND2* and *Cyt b* using primers and cycling parameters outlined in Peters et al. (2022). Samples with a low DNA yield underwent a primerless PCR process, also outlined in Peters et al. (2019) prior to amplification. PCR was conducted using illustra™ Hot Start Mix RTG PCR Beads (Cytiva; Sheffield) with a final volume of 25 μL containing ~1.75 units of recombinant PuReTaq™ DNA polymerase, 1.65 μg of Hot Start Activator, 10 mM Tris–HCl (pH 9.0 at room temperature), 50 mM KCl and 1.5 mM MgCl_2_, 200 μM of dNTP's, BSA and reaction buffer, 8 μL of DNA template and a negative control. PCR purification was carried out using the QIAquick PCR & Gel Clean‐up Kit as per the manufacturer's protocols (QIAGEN Inc., Crawley). Samples were visualised on a 1% agarose gel (Thermo Fisher Scientific, Waltham) using a BioRad Gel Doc™ EZ Imager and quantified using Image lab 4.0 software (Bio‐Rad Laboratories 2017). Samples were prepared and submitted to Eurofins Genomics following the Mix2Seq kit (Eurofins Genomics, Luxembourg) instructions for sequencing using Sanger sequencing methods. Sequence assembly was performed using Sequencher 5.4.6 (Gene Codes Corporation, 2017).

### 2.3 Molecular sexing

Samples were sexed using the universal avian sexing primers P8 and P2 (Griffiths et al., 1998) and cycling parameters outlined in Çakmak et al. (2017). The PCR product was visualised on a 3% agarose gel to determine sex based on banding pattern of one band for males and two bands for females (Çakmak et al., 2017; Griffiths et al., 1998; Morinha et al., 2012). Sexing was performed in triplicate to ensure accuracy (Chang et al., 2008).

### 2.4 Analysis of mtDNA genetic diversity

The final sample set used for each site was as follows: Mt Hartman (*n* = 18: feathers (*n* = 16); eggshells [*n* = 2]) and Perseverance (*n* = 12: feathers (*n* = 12); Table 1), representing approximately 18% of the total Grenada Dove population. Genetic analysis were conducted using the PopGenome and Pegas packages (Paradis, 2010; Pfeifer et al., 2014) in R version 3.6.2 (R Core Team, 2021). To assess genetic diversity, biallelic sites and mutation type were identified, haplotypes were determined using the haplotype count function and haplotype (*h*) and nucleotide (π) diversity were calculated. Sequences with low average quality score *ND2* (*n* = 7) and *Cyt b* (*n* = 1) were removed from mtDNA genetic analyses.

**TABLE 1:** Number of samples, samples type and collection information for the samples included in this study. Additional information can be found in Appendix S2.

Site	Sample type	Year	Number collected
Mount Hartman	Eggshell	2018	1
Mount Hartman	Eggshell	2017	1
Mount Hartman	Body feather	2015	2
Mount Hartman	Body feather	Unknown	1
Mount Hartman	Body feather	2015	4
Mount Hartman	Body feather	2017	3
Mount Hartman	Body feather	2018	1
Mount Hartman	Body Feather	Unknown	3
Mount Hartman	Body feather	2014	1
Mount Hartman	Body feather	Unknown	1
Perseverance	Body feather	2017	6
Perseverance	Body feather	2015	2
Perseverance	Body feather	2017	3
Perseverance	Secondary feather	2017	1

## 3 RESULTS

### 3.1 mtDNA genetic diversity

**FIGURE 2:**
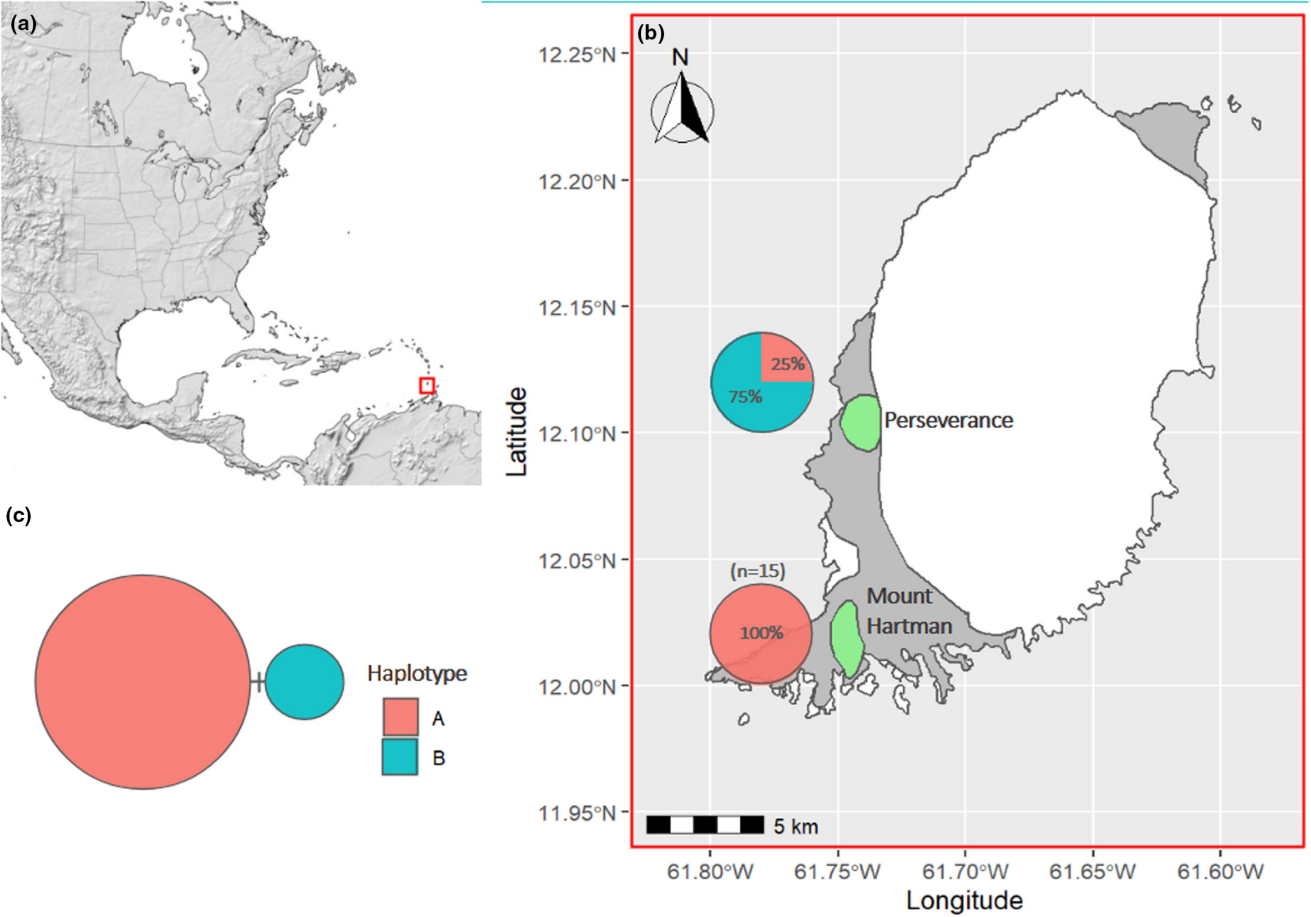
(a) Map of the Caribbean within the Americans, with the island of Grenada highlighted by the red square, (b) Grenada Dove haplotype distribution among sampling localities on Grenada with historic range in grey and extant range in green and (c) haplotype network with size proportional to the number of samples and a bar to represent the single transitional mutation between the two haplotypes.

A total of 1751 bps of mtDNA (*n* = 23) were amplified which is comprised of 965 bps of *ND2* and 786 bps of *Cyt b*. Two unique haplotypes were identified using the 1751 bps combined gene regions with a haplotype diversity (*h*) of 0.4 and a nucleotide diversity (*π*) of 0.00024. The most common haplotype (A = 73.9%) was found at both sites, and the second haplotype (B = 26.1%) was found only in Perseverance (Figure 2).

Variation within the 965 bp of *ND2* (*n* = 23) included one single transitional polymorphism at the 882 bp position, and as such, two haplotypes were present (Table 2). No variation was detected within the single haplotype identified within the 786 bps of the *Cyt b* gene (*n* = 29).

**TABLE 2:** Number of samples, haplotypes and polymorphic sites for *ND2* and *Cyt b* individually along with the concatenated data for both mtDNA genes.

Gene	Sample size (*n*)	No. of haplotypes	No of polymorphic sites
ND2 (965 bps)	23	2	1
Cyt b (786 bps)	29	1	0
mtDNA (1751 bps)	23	2	1

### 3.2 Population comparison

Observed within population diversity using the 1751 bp combined gene regions (*n* = 23) was higher at Perseverance (*h* = 0.43, π = 0.00024) in comparison to Mt Hartman which showed no variation in any of the samples from that site (Table 3). Mt Hartman (*n* = 15) was comprised solely of haplotype A: 15 (100%) whereas Perseverance (*n* = 8) comprised of haplotype A: two (25%) and haplotype B: six (75%) (Figure 2).

**TABLE 3:** Genetic diversity indices based on 1751 bp of mtDNA (*ND2* and *Cyt b*) for each population.

Site	No. of Individuals (*n*)	No. of haplotypes	No. of unique haplotypes	Haplotype composition	No of polymorphic sites	Haplotype diversity (*h*)	Nucleotide diversity (*π*)
Perseverance	8	2	1	A: 2(25%) B: 6(76%)	1	0.43	0.00024
Mt Hartman	15	1	0	A: 15(100%) B: 0(0%)	0	0	0
Overall	23	2	—	A: 17(73.9%) B: 6(26.1%)	1	0.4	0.00023

### 3.3 Molecular sexing

Molecular sexing was carried out for all the samples and indicated the sample set was comprised of five females and 18 males (Mt Hartman: females = 5, males = 11; Perseverance: females = 0, males = 7) with the sex unknown for the remaining samples due to amplification failure for this marker.

## 4 DISCUSSION

This study is the first to evaluate genetic diversity in populations of the critically endangered Grenada Dove and has revealed extremely low haplotype diversity based on the examination of two mitochondrial genes obtained from non‐invasively collected samples. The two extant sites that constitute the entire range of the species are likely isolated from each other, as shown by the unique haplotype found in the West but not the Southwest population. Molecular sexing revealed a male sex bias in the samples used in this study. Given the estimated population size of around 160 individuals (Rivera‐Milán et al., 2015), along with isolated populations, this species is at increased risk of inbreeding depression and further loss of genetic diversity due to genetic drift, which can lead to decreased fitness and survival (Brekke et al., 2010; Houston et al., 2015; Price et al., 2015; Wright et al., 2008). Therefore, management interventions at a population level are required to prevent further loss of genetic variation in the future (Kahilainen et al., 2014; Price et al., 2015).

### 4.1 mtDNA genetic diversity

The number of haplotypes (2) and nucleotide polymorphisms (1) obtained in this investigation are low in comparison to similar mtDNA studies carried out on other *Columbidae* species for example: Zenaida dove *Zenaida aurita* 11 haplotypes (Monceau et al., 2013); Japanese wood pigeon *Columba janthina* 27 haplotypes (Seki et al., 2007); and Eurasian collared dove *Streptopelia decaocto* 52 haplotypes (Bagi et al., 2018). Our results are similar to that found for another Endangered Caribbean island endemic species, the Bahama oriole *Icterus northropi*, which reported a haplotype number of *n* = 2 and haplotype diversity of *h* = 0.40 for the smaller southernmost subpopulation of this species; however, the nucleotide diversity value reported (π = 0.067) was much higher than for the Grenada Dove (Price et al., 2015). While every precaution has been made to ensure data was collected from different individuals, due to the non‐invasive and opportunistic nature of the sample collection method, sampling of the same individual may have occurred. Multiple replicates from the same individual could lead to inaccuracies such as underestimation of genetic diversity (Bashalkhanov et al., 2009; Jackson et al., 2016; Schultz et al., 2021). However, as the samples were filtered both temporally and spatially, combining this with molecular sexing data, the number of repeated samples from individuals is likely to be minimal. Despite the challenges of using non‐invasively and opportunistically collected samples, which are often considered unsuitable for genetic analysis, we were able to use such samples for downstream conservation genetic applications in a wild bird population to provide evidence suggesting that the Grenada Dove may have low mitochondrial genetic diversity.

### 4.2 Population comparison

This study provides evidence of genetic differences between the Grenada Dove from two different sites: Perseverance in the West and Mt Hartman Estate in the Southwest of the island. One haplotype (Haplotype A: Mt Hartman = 100%, Perseverance = 25%) was shared between both locations, whereas the other haplotype identified (Haplotype B: Mt Hartman 0%, Perseverance = 75%) was unique to Perseverance. Higher haplotype and nucleotide diversities were observed within the samples at Perseverance (*n* = 8) compared to Mt Hartman (*n* = 15), which had no genetic variation despite a larger sample size and larger population (Rusk, 2009, 2017). This highlights the important role of the dry forest at Perseverance as the location of the highest genetic diversity for this species, particularly as Perseverance is at the smaller of the two populations (Rusk, 2017). Overall, these data indicate that there is genetic divergence between geographically subdivided populations. However, caution is recommended as the nucleotide difference among haplotypes is just one polymorphic variation which potentially affects the interpretation through possible overestimation of the divergence (Bhatia et al., 2013). Both the haplotype unique to Perseverance and the genetic divergence between the two localities suggest that these populations are now isolated from each other. As there was also a haplotype shared between both populations, these data support the belief that these regions were historically connected and have since become isolated (Rusk, 2017). However, it is important to note that differences in rates of evolution between and within gene regions may be reflected in the observations made from our results (Johnson & Sorenson, 1998; Nabholz et al., 2009). While all mitochondrial protein coding genes have similarly high rates of substitution, *ND2* is thought to be the third most variable gene (Johnson & Sorenson, 1998); hence, a polymorphism was detected in this gene and none were detected in *Cyt b*. As such, our results demonstrate that *ND2* could provide information concerning more recent gene flow, whereas *Cyt b* may be more informative for historical isolation.

Population isolation is a threat to this species given its already small numbers (Rusk, 2009) as small isolated populations are at risk of losing genetic diversity over time through stochastic genetic drift (Fraser, 2017). If, as our results (representing approximately 18% of the total population) indicate, there is little to no gene flow between the two populations; this reduces potential for mate choice and increases the probability of mating between related individuals (Jensen et al., 2007; Price et al., 2015). Such low gene flow increases the risk of extinction to small populations whose genetic diversity can erode as a result of inbreeding (Kahilainen et al., 2014). Future genetic studies should include nuclear regions to discern the extent to which the Grenada Dove populations are isolated, and help assess levels of inbreeding (Ewing et al., 2008; Hagen et al., 2011; White et al., 2014). Outstanding genetic information pertaining to, for example, population structure, inbreeding depression, genetic relatedness, adaptive loci and levels of heterozygosity, could be obtained via the collection of microsatellites (Friesen et al., 2006; Hagen et al., 2011; Reynolds et al., 2015) and restriction site associated DNA (RAD) sequencing (Dierickx et al., 2015, 2019; Ryan, 2021) data which, while potentially requiring higher sample quality (Carroll et al., 2018), would further aid the conservation management of the Grenada Dove.

### 4.3 Molecular sexing

Of the samples which could accurately be assigned a sex, the sample set comprised 18 males and five females. The samples assigned as female consisted of only haplotype A. The samples assigned as male consisted of 61% haplotype A and 28% haplotype B. All samples from females were collected at Mt Hartman, whereas 66.6% of the male samples were collected from Mt Hartman and 33.33% from Perseverance. While this may be indicative of a male skewed sex ratio, caution is needed because of the non‐invasive, opportunistic sample collection method which may have resulted in the presence of duplicate samples. Due to the paucity of information regarding the breeding ecology of this species, such as the effects of age, sex, climate and breeding cycles on moult (Brown et al., 2002; Kiat et al., 2019; Leeson & Walsh, 2007), we were unable to interpret whether bias could have been generated by the sample collection method. Future studies could employ a more robust sampling strategy and consider sampling across the whole year to mitigate against biases if factors such as breeding season influence male moult (Kiat et al., 2019; Leeson & Walsh, 2007). A 1:1 adult sex ratio (ASR) has often been assumed for the Grenada Dove even though it has been acknowledged that there is potential for a male‐sex bias (Bolton et al., 2015; Rivera‐Milán et al., 2015). A male skew is not uncommon in pigeons and doves as evidenced by the pink pigeon (Bunbury, 2006), scaled pigeon *Patagioenas speciose* and plain‐breasted ground dove *Columbina minuta* (Bosque & Pacheco, 2019). Skewed sex ratios may not be the result of unequal production of the sexes at birth but could be the result of differential adult survival rates where one sex is at higher risk of predator‐based mortality or more susceptible to parasites and disease (Bosque & Pacheco, 2019; Székely et al., 2014). There have been a number studies on ASR in bird populations which have revealed, for example, that a male skew is frequently seen in threatened species (Donald, 2007) as well as species with small ranges and island species (Venables & Brooke, 2015). The majority of studies, however, have been carried out on species in temperate latitudes and so sex ratio information for birds at tropical latitudes is largely unknown (Bosque & Pacheco, 2019). The results collected in this study reflect this suspected male skew which, if correct, could mean that the reported population size for this species may be lower than current estimates, which are extrapolated from data on calling males (Bolton et al., 2015). If accurate, the low maternal genetic diversity in this data could be influenced by this male sex bias (Houston et al., 2015). Carrying out genetic diversity assessment using nuclear markers, which are inherited from both parents, would avoid these issues; however, this was not possible in this study because of the type and degraded nature of the samples (Peters et al., 2019). ASRs are a key component in understanding species' demography and biology (Bosque & Pacheco, 2019; Donald, 2007; Székely et al., 2014) and therefore further investigation into the sex ratio, such as the banding programme proposed in Bolton et al. (2015), could be critical for the survival of this critically endangered species.

### 4.4 Conservation implications

To conserve species with fragmented or isolated populations, an understanding of genetic diversity is recommended before undertaking any management interventions (Frankham et al., 2017; vanDyke, 2008). The data from this study revealed unique genetic variation at one locality (Perseverance) suggesting additional habitat protection at this site is important for conserving genetic diversity in this species (Tarr & Fleischer, 1999). Re‐introduction of the Grenada Dove to dry forest in its northern historic range, using individuals from both Mt Hartman and Perseverance, could also be beneficial as geographically separate populations are not only important for promoting genetic diversity (du Plessis et al., 2019; Gregory et al., 2012; Major et al., 2021) but also in safeguarding against natural disasters such as hurricanes. Given recent habitat loss on Grenada, availability of suitable habitat on the island for establishing new populations is limited and would likely result in additional isolated populations (Prugh et al., 2008; Rusk et al., 2008). Additional separate populations can be a useful conservation approach, serving as insurance populations for species with small numbers and limited occurrence, should other populations be threatened (Major et al., 2021). Establishing new populations using a small number of founder individuals is a viable approach when considering species of conservation concern (Grueber et al., 2017). This is not uncommon in island species and populations have been known to successfully recover from a very small number of individuals (Copsey et al., 2018), for example, the Pink Pigeons went from nine or 10 individuals in 1990 to 446 in 2011 (Swinnerton et al., 2004), the Echo Parakeet *Psittacula eques* went from as low as 12 individuals in the 1990s to more than 500 in 2010 (Raisin et al., 2012) and the Rodrigues Warbler *Acrocephalus rodericanus* started with approximately 17 individuals in 1979 and grew to more than 3000 in 2010 (Copsey et al., 2018; Showler et al., 2002). Additional isolated populations may be useful for rescue measures and to protect against localised extinction (du Plessis et al., 2019; Frankham et al., 2019; Grant et al., 2001); however, reliable information and regular assessment of genetic variability are essential for conservation decisions and to monitor whether new populations are successfully established (Gregory et al., 2012).

We provide evidence of population isolation for the Grenada Dove. Conventionally, recommendations to increase gene flow between isolated populations would be through habitat connectivity (Frankham et al., 2019; Lindsay et al., 2008). However, this is unrealistic in this case due to the expansive human development which separates the two sites. Augmentation of gene flow may be more easily facilitated on this occasion by translocation of individuals with haplotype B between the populations; however, translocations should only be considered alongside sufficient in‐situ management (Dolman et al., 2015). Despite evidence of low genetic diversity at each population, such genetic rescue measures were successfully used on isolated populations of an island endemic species, the South Island Robin *Petroica australis* (Heber et al., 2012). Heber et al. (2012) used highly inbred populations as donors to rescue two isolated populations. Translocations of 31 female robins led to an increase in heterozygosity, immunocompetence and juvenile survival in individuals crossed between the two populations in comparison to the those crossed within each population. We would recommend, once appropriate and effective demographic rescue and in situ management measures are in place, that genetic rescue measures be implemented with the aim of augmenting genetic diversity at Mt Hartman and reducing the risk of extinction at this site (Frankham et al., 2019; Jackson et al., 2022).

## 5 CONCLUSION

This study has provided evidence of low mitochondrial genetic diversity, genetically isolated populations and a potentially male skewed sex ratio in the Grenada Dove obtained from non‐invasively collected samples. Two haplotypes were identified, the most common haplotype was observed at both sites and the other was unique to Perseverance. This suggests historical connectivity and subsequent isolation of these two populations. These data suggest that the population at Perseverance is the most genetically diverse of the two highlighting the importance of this forest in maintaining the genetic diversity of this species. A thorough assessment of genetic diversity and further long‐term monitoring alongside in situ management could therefore be important in future conservation decisions in order to maximise genetic diversity in this species and avoid inbreeding over time. In the light of the findings of this study, we recommend increased habitat protection to maintain the two extant populations, establishing a new population to increase genetic diversity and mitigate the risk of catastrophic events, and augmentation of gene flow by translocation between the two extant populations in order to increase genetic resilience and diversity, with the ultimate aim of securing the long‐term survival of this critically endangered species.

## AUTHOR CONTRIBUTIONS

**Catherine Peters:** Conceptualization (lead); data curation (lead); formal analysis (lead); funding acquisition (equal); investigation (lead); methodology (lead); project administration (lead); validation (lead); visualization (lead); writing – original draft (lead); writing – review and editing (lead). **Matthew Geary:** Conceptualization (equal); formal analysis (supporting); investigation (supporting); methodology (supporting); project administration (supporting); supervision (lead); visualization (supporting); writing – original draft (supporting); writing – review and editing (supporting). **Charlotte Hosie:** Project administration (supporting); supervision (supporting); writing – review and editing (supporting). **Howard Nelson:** Conceptualization (supporting); funding acquisition (supporting); project administration (supporting); supervision (supporting); writing – review and editing (supporting). **Bonnie Rusk:** Conceptualization (supporting); writing – review and editing (supporting). **Anna Muir:** Conceptualization (supporting); formal analysis (supporting); investigation (supporting); methodology (supporting); project administration (supporting); supervision (supporting); validation (supporting); visualization (supporting); writing – original draft (supporting); writing – review and editing (supporting).

## FUNDING INFORMATION

This work was supported by the University of Chester Biological Sciences department.

## CONFLICT OF INTEREST STATEMENT

The authors have no relevant financial or non‐financial interests to disclose.

## Supporting information


Appendix S1.

Appendix S2.

## Data Availability

The data sets comprised of DNA sequence data generated using non‐invasive feather and eggshell samples from the Grenada Dove for two gene regions: Cyt b and ND2 generated during and analysed during this study are available from the corresponding author on reasonable request or via this link: https://doi.org/10.5061/dryad.dv41ns252.
