# Supplementary material for: Non‐invasive sampling reveals low mitochondrial genetic diversity for an island endemic species: The critically endangered Grenada Dove Leptotila wellsi
Source: Ecol Evol. 2023 Nov 23;13(11):e10767. doi: 10.1002/ece3.10767 (PMC10667608; doi:10.1002/ece3.10767)
Supplement: Supplementary file 1 — Appendix S1. [file ECE3-13-e10767-s001.pdf]

Online Resource 1:

Species-specific primers were designed based on a unique region of Cytochrome Oxidase I (*COI*) sequence obtained from specimens provided by the Head of the Forestry and National Parks Department, Mr Anthony Jeremiah, and Forest Ranger, Mr Doland Francis (Peters *et al.* 2022). The designed primers: LW4_F: GTTATACCAATCATGATTGGG and LW4_R: GTTAATGGCGGTTGTAATAAAG targeted a 261 bp region of *COI* and were tested against seven species, including 3 closely related species found on Grenada, to ensure amplification of Grenada Dove DNA only (Figure ESM1; Table ESM1). Optimised PCR cycling parameters were as follows: initial denaturation at 95 °C for 5 min, 45 cycles of 95 °C for 30 s, 58 °C for 30 s, 72 °C for 60 s and a final extension at 72 °C for 5 min. All non-invasively collected samples were processed using this primer set. Demographic and biological information can be used to assist individual identification (Johnson *et al.* 2007a; Russello *et al.* 2015), therefore temporal and spatial information provided with the samples - site location, GPS information and sample collection dates – were used along with molecular sexing data in an effort to distinguish between different individuals (Online Resource 2).

Using standard sequence identification methods, sequenced samples were queried in NCBI's Basic Local Alignment Search Tool (BLAST®) nucleotide database to confirm species identification (Jarman *et al.* 2004; Ross *et al.* 2008; Ovaskainen *et al.* 2010; Zhuang *et al.* 2012; Price *et al.* 2015). A BLAST® search was also conducted for each sample against previously obtained Grenada Dove samples (Peters *et al.* 2022). The sequences obtained for this study were consistent with previously published sequences for *Leptotila* (*ND2*: >94.56% Per. Ident with 882 total score for *Leptotila plumbeiceps* Accession Number: HQ993544.1; *Cyt b* : >95.56% Per. Ident with 970 total score for *Leptotila cassini* Accession Number: HQ993505.1 (Johnson and Weckstein 2011)). This indicates the DNA fragments amplified in this study represent the intended target species, which is the only *Leptotila* resident on Grenada (Peters *et al.* 2022).

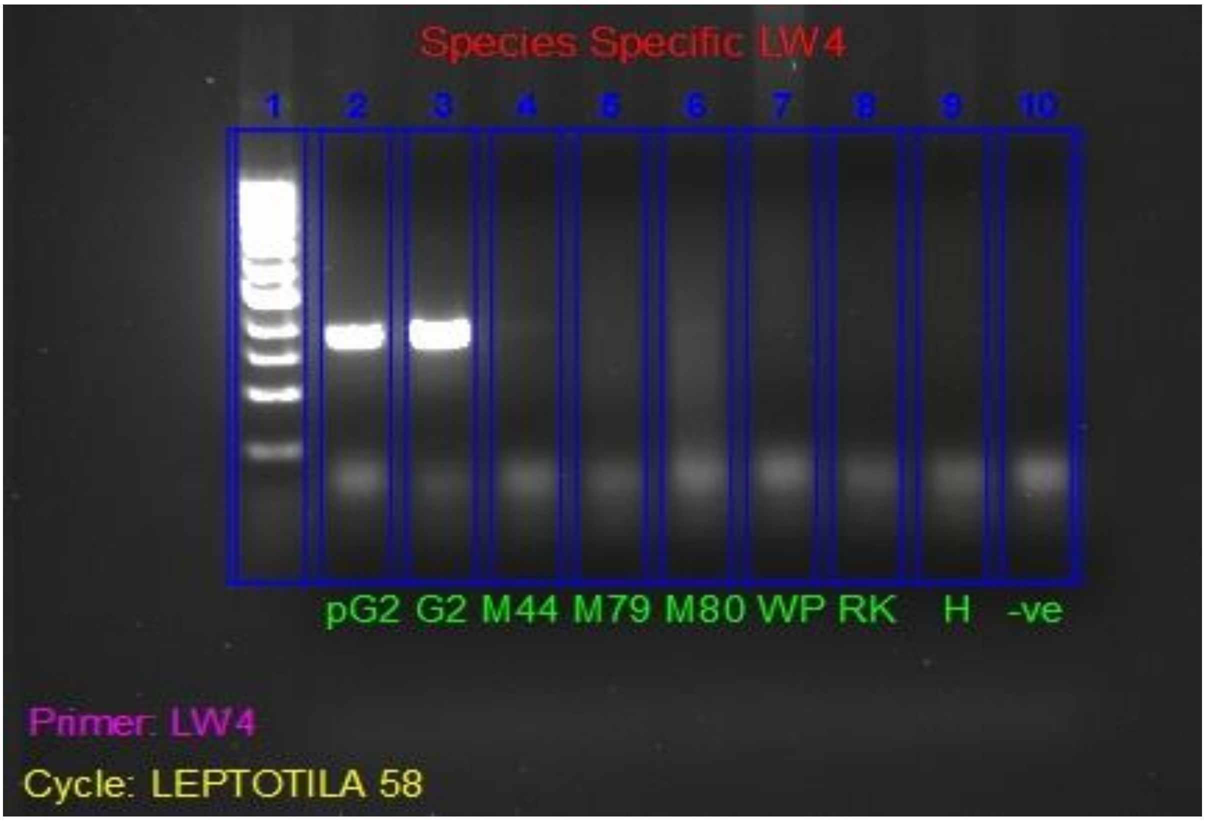

Figure ESM1: Gel (2% w/v agarose) image showing species specific primer optimisation displaying successful amplification of target species only with corresponding sample information shown in table ESM1.

Table ESM1: Species specific primer optimisation sample information for corresponding gel image above.			
Lane	Sample	Sample Source	Species
2	pG2	Forestry and National Parks Department of Grenada	*Leptotila wellsi*
3	G2	Forestry and National Parks Department of Grenada	*Leptotila wellsi*
4	M44	Forestry and National Parks Department of Grenada	*Zenaida aurita**[1]
5	M79	Forestry and National Parks Department of Grenada	*Patagioenas leucocephala**[2]
6	M80	Forestry and National Parks Department of Grenada	*Petrophassa rufipennis* *[3]
7	WP	University of Chester	*Columba palumbus*
8	RK	University of Chester	*Milvus milvus*
9	H	University of Chester	*Homo sapiens*

*NCBI's Basic Local Alignment Search Tool (BLAST®) used to predict species identification from non-invasively collected samples

[1] *Zenaida aurita* - Genbank: accession number AF182704.1; total score 1681; Per. Ident 98.24%

[2] *Patagioenas leucocephala* - Genbank: accession number JQ175689.1; total score 989; Per. Ident 96.77%

[3] *Petrophassa rufipennis* - Genbank: accession number KU194386.1; total score 1203; Per. Ident 92.19%

References

Jarman SN, Deagle BE, Gales NJ (2004) Group-specific polymerase chain reaction for DNA-based analysis of species diversity and identity in dietary samples. Mol Ecol 13:1313–1322. https://doi.org/10.1111/j.1365-294X.2004.02109.x

Johnson JA, Burnham KK, Burnham WA, Mindell DP (2007) Genetic structure among continental and island populations of gyrfalcons. Mol Ecol 16:3145–3160. https://doi.org/10.1111/j.1365-294X.2007.03373.x

Johnson KP, Weckstein JD (2011) The Central American land bridge as an engine of diversification in New World doves. J Biogeogr 38:1069–1076. https://doi.org/10.1111/j.1365-2699.2011.02501.x

Ovaskainen O, Nokso-Koivisto J, Hottola J, et al (2010) Identifying wood-inhabiting fungi with 454 sequencing - what is the probability that BLAST gives the correct species? Fungal Ecol 3:274–283. https://doi.org/10.1016/j.funeco.2010.01.001

Peters C, Geary M, Nelson HP, et al (2022) Phylogenetic placement and life history trait imputation for Grenada Dove Leptotila wellsi. Bird Conserv Int 1–11. https://doi.org/10.1017/S0959270922000065

Price MR, Person C, Hayes WK (2015) Geographic variation and genetic structure in the Bahama Oriole (Icterus northropi), a critically endangered synanthropic species. PeerJ 2015:1–16. https://doi.org/10.7717/peerj.1421

Ross HA, Murugan S, Li WLS (2008) Testing the reliability of genetic methods of species identification via simulation. Syst Biol 57:216–230. https://doi.org/10.1080/10635150802032990

Russello MA, Waterhouse MD, Etter PD, Johnson EA (2015) From promise to practice: pairing non-invasive sampling with genomics in conservation. PeerJ 3:e1106. https://doi.org/10.7717/peerj.1106

Zhuang Y, Zhou X, Wang S (2012) Gapped BLAST and PSI-BLAST: a new generation of protein database search programs. Plant Syst Evol 298:3389–3402. https://doi.org/10.2503/jjshs.58.977

TITLE: Non-invasive sampling reveals low mitochondrial genetic diversity an island endemic species: the Critically Endangered Grenada Dove *Leptotila wellsi*

Authors: Catherine Peters (Corresponding author: c.peters@chester.ac.uk; Conservation Biology Research Group, Department of Biological Sciences, University of Chester, Chester, CH1 4BJ, UK)**,** Matthew Geary, Charlotte Hosie, Howard Nelson, Bonnie Rusk, Anna Muir.
